# Angiogenesis in rheumatoid arthritis: the role of fut 1

**DOI:** 10.1186/ar3584

**Published:** 2012-02-09

**Authors:** Alisa Erika Koch

**Affiliations:** 1 Department of Veteran's Affairs, Ann Arbor, MI USA and University of Michigan, MI, USA

## 

In rheumatoid arthritis (RA), targeting the vasculature may be beneficial to control the disease. Endothelial cells lining blood vessels are involved in a variety of functions in inflammation, including recruitment of leukocytes and cellular adhesion, antigen presentation, coagulation, cytokine production and angiogenesis. Angiogenesis, the growth of new vessels, is important for the proliferation of the rheumatoid synovial tissue pannus where these vessels also serve as a conduit for cells entering the inflamed synovium from the blood.

We have shown before that the endothelial adhesion molecule E-selectin, in soluble form, mediates angiogenesis via its endothelial receptor sialyl Lewis^x ^on adjacent endothelium [1]. We have used human RA synovial tissues to produce an antibody detecting related molecules, Lewis^y^/H-5-2, which are mainly known as blood group antigens but are also found on endothelium in select organs such as skin, lymph node and synovium, but not most other endothelium. This antigen is rapidly upregulated on endothelium *in vitro *in response to stimuli such as tumor necrosis factor-alpha, that is present in the RA joint. Additionally, this antigen is upregulated on RA vs. normal synovial endothelial cells, and in soluble form is upregulated in RA synovial fluid vs. osteoarthritic synovial fluid. In soluble form, Lewis^y^/H-5-2 mediates angiogenesis, cell adhesion via intercellular adhesion molecule-1, and monocyte recruitment.

Fucosyl transferases (fut1 and fut2) are enzymes that control the synthesis of Lewis^y^/H-5-2. We have examined fut1 deficient mice to determine if fucosylation is important in angiogenesis and arthritis. Fut1 gene deficient mouse endothelial cells did not form endothelial sprouts on Matrigel *in vitro *to the same extent as wild type mouse endothelial cells. Moreover, the fut1 gene deficient mice were resistant to the development of angiogenesis in the Matrigel plug and sponge granuloma angiogenesis models *in vivo*. In terms of arthritis development, the Lewis^y^/H-5-2 gene deficient mice were resistant to development of K/BxN arthritis. Moreover, the harvested joints of these mice had decreased monocyte chemoattractant protein-1/CCL2 and interleukin-1 compared to wild type littermates, indicating that some inflammatory mediators were downregulated when fut1 was absent. These experiments suggest that futs may be important in the development of angiogenesis and inflammatory arthritis and that they may serve as novel targets in RA therapy.
